# Validation of the trauma mortality prediction scores from a Malaysian population

**DOI:** 10.1186/s41038-017-0102-z

**Published:** 2017-12-22

**Authors:** Jih Huei Tan, Henry Chor Lip Tan, Nur Azlin Md Noh, Yuzaidi Mohamad, Rizal Imran Alwi

**Affiliations:** 1 General Surgery Department, Hospital Sultanah Aminah, Johor Bahru, Malaysia; 2 Pusat Perubatan Universiti Kebangsaan Malaysia, Cheras, Malaysia; 3 Clinical Research Centre, Hospital Sultan Ismail, Johor Bahru, Malaysia

**Keywords:** Trauma scoring system, Prediction model, Injury grading, Southeast Asia, New Injury Severity Score, Revised Trauma Score, Trauma and Injury Severity Score

## Abstract

**Background:**

Well-known trauma mortality prediction scores such as New Injury Severity Score (NISS), Revised Trauma Score (RTS), and Trauma and Injury Severity Score (TRISS) have been externally validated from high-income countries with established trauma databases. However, these scores were never used in Malaysian population. In this current study, we attempted to validate these scoring systems using our regional trauma surgery database.

**Methods:**

A retrospective analysis of the regional Malaysian Trauma Surgery Database was performed over a period of 3 years from May 2011 to April 2014. NISS, RTS, Major Trauma Outcome Study (MTOS)-TRISS, and National Trauma Database (NTrD)-TRISS scores were recorded and calculated. Individual scoring system’s performance in predicting trauma mortality was compared by calculating the area under the receiver operating characteristic (AUC) curve. Youden index and associated optimal cutoff values for each scoring system was calculated to predict mortality. The corresponding positive predictive value, negative predictive value, and accuracy of the cutoff values were calculated.

**Results:**

A total of 2208 trauma patients (2004 blunt and 204 penetrating injuries) with mean age of 36 (SD = 16) years were included. There were 239 deaths with a corresponding mortality rate of 10.8%. The AUC calculated for the NISS, RTS, MTOS-TRISS, and NTrD-TRISS were 0.878, 0.802, 0.812, and 0.848, respectively. The NISS score with a cutoff value of 24, sensitivity of 86.6% and specificity of 74.3%, outperformed the rest (*p* < 0.001). Mortality was predicted by NISS with an overall accuracy of 75.6%; its positive predictive value was at 29.02% and negative predictive value at 97.86%.

**Conclusion:**

Amongst the four scores, the NISS score is the best trauma mortality prediction model suited for a local Malaysian trauma population. Further validation with multicentre data in the country may require to ascertain the finding.

## Background

Traumatic injuries are a significant burden to our healthcare system especially in the lower income countries [1]. A lack of modernized public transportation, lax road traffic law enforcement, and wide geographical landscape lead to a poorer outcome in the majority of traumatic cases. Therefore, by setting up a trauma unit within a tertiary hospital may improve the outcomes of traumatic injuries [2].

With the aim of improving trauma treatment outcomes, the first Malaysian trauma surgery unit was developed at Hospital Sultanah Aminah (the study site) in 2011 [3]. The unit is led by two resident trauma surgeons and two trauma nurses to organize work effectively. This pioneer trauma center receives trauma patients mainly from the southern region of Peninsular Malaysia. Treatment is provided for more than 750 patients per year. The initial census found that the majority of the trauma patients (53%) were in the severely injured category. This group of patients had an average Injury Severity Score (ISS) of 15 and above [4].

Differences in trauma treatments in Malaysia and developed nations contribute to a variable pattern of trauma survivability. The lack of having a specialized trauma team to manage trauma cases is one of the differences in our trauma care services. In our local setting, the medical personnel handles a wide range of medical and surgical emergencies in contrast to developed nations whereby a specialized trauma team is available to manage trauma cases. Invariably, this reduces survivability as evidenced by several studies which concluded that by having a specialized trauma team may improve outcomes of mortality [5]. In addition, the lack of air transport delays the treatment of trauma patients in geographically inaccessible areas. Patients requiring immediate evacuation may be delayed due to the usage of land transportation to tertiary center. Other differences include a mixture of operating theaters and intensive care setup of surgical and medical patients. A recent publication by Klein et al. and Lee et al. has shown that the presence of a surgical intensive care unit (ICU) or trauma intensivist reduces the patient’s ICU stay, ventilator days, and reduced pulmonary complications [6, 7]. From these three differences observed, it is of upmost importance to triage and prognosticate patients in terms of survivability. One of the methods to triage patient according to risk is by using mortality risk prediction scores.

According to Bowser et al., mortality prediction models is defined as a discriminant analysis using multifactorial methods for discriminating between dichotomous outcomes of survivors and non-survivors [8]. The use of trauma prediction models can be found as early back to 1974 of the ISS by Baker et al. [9]. Amongst the other commonly used scores are New Injury Severity Score (NISS), Revised Trauma Score (RTS), and Trauma and Injury Severity Score (TRISS). Historically, NISS is an anatomic score developed by Sir William Osler in 1997 [10]. NISS is calculated as the sum of the squares of the top three scores regardless of body region [10]. Subsequently, the RTS score which incorporated physiological parameters was devised by Champion et al. [11] TRISS is a combination of both the anatomic and physiologic parameters which utilizes the specific coefficients derived from multiple regression analysis of the Major Trauma Outcome Study (MTOS) database [12]. These three scoring systems are commonly being used by practitioners worldwide due to its familiarity and validated in numerous countries [13].

All of three trauma prediction models (NISS, RTS, and TRISS) received external validation from trauma centers in developed nations [14-17]. These scores were never validated in a Malaysian population. Considering the unique differences described and the lack of external validation of these scores in a developing nation, the aim of this study is to compare the predictability of NISS, RTS, MTOS-TRISS, and National Trauma Database (NTrD)-TRISS to determine trauma mortality in a Malaysian population.

## Methods

This is a retrospective data analysis of all trauma admissions within a 3-year period from May 2011 to April 2014. Data was collected from a prospectively maintained trauma registry at the trauma center of Hospital Sultanah Aminah, Johor Bahru. Patient records were screened, and only trauma patients aged more than 13 years old and managed in our center were included. Patients aged 13 years and below were excluded as they will be managed by a specialized pediatric surgery team. In addition, all patients with traumatic pathological conditions (e.g., pathological fractures resulting from malignancy), injuries as a result of degenerative changes, hanging, drowning, burns and envenomation, and isolated head and isolated skeletal fractures without hemodynamic compromise were also excluded.

All trauma patients were assessed with the NISS, RTS, MTOS-TRISS [12], and NTrD-TRISS [18] which were calculated retrospectively by two trained trauma nurses. The data collected was verified by trauma surgeons’ prior data analysis.

Both MTOS-TRISS and NTrD-TRISS give the probability of survival (*P*_*s*_).

MTOS-TRISS uses the coefficients from the original MTOS study [12]. The calculation has two separate specifications for adults (≥ 15 years of age): (i) for injuries sustained from a blunt mechanism, and (ii) for injuries sustained from a penetrating mechanism. Specification (i) is also universally applied to children (< 15 years of age), regardless of the mechanism of injury [19].

The *P*_*s*_ for any one patient can be estimated from: *P*_*s*_ = 1/ (1 + *e* ^*−b*^), where for adults with blunt mechanism trauma,

$$ b=\left(-0.4499\right)+(0.8085)\left(\mathrm{RTS}\right)+\left(-0.0835\right)\left(\mathrm{ISS}\right)+\left(-1.7430\right)\left(\mathrm{AgeIndex}\right) $$

and for adults with penetrating mechanism trauma,

$$ b=\left(-2.5355\right)+(0.9934)\left(\mathrm{RTS}\right)+\left(-0.0651\right)\left(\mathrm{ISS}\right)+\left(-1.1360\right)\left(\mathrm{AgeIndex}\right) $$

ISS has values from 0 to 75; AgeIndex is 0 if patient age is 15 to 54 years and 1 if patient age is ≥ 55 years. The RTS is given by the following equation containing 3 variables: respiratory rate (RR), systolic blood presure (SBP) and Glasgow Coma Scale (GCS). The parameters are converted to score (0, 1, 2, 3, or 4),

$$ \mathrm{RTS}=\left(0.2908\times \mathrm{RRscore}\right)+\left(0.7326\times \mathrm{SBPscore}\right)+\left(0.9368\times \mathrm{GCSscore}\right). $$

Table 1 shows categorization of variables for RTS calculation.

**Table 1:** Categorization of variables for Revised Trauma Score (RTS) calculation

Variable	Value	Score
Respiratory rate (breaths/min)	10–29	4
	> 29	3
	6–9	2
	1–5	1
	0	0
Systolic blood pressure (mmHg)	> 89	4
	76–89	3
	50–75	2
	1–49	1
	0	0
Glasgow Coma Scale	13–15	4
	9–12	3
	6–8	2
	4–5	1
	3	0

NTrD-TRISS uses the coefficients from a study using Malaysia national trauma database [18]. Similarly, the *P*_*s*_ for any one patient can be estimated from *P*_*s*_ = 1/ (1 + *e* ^−*b*^). However, the calculations of “*b*” were same for blunt and penetrating mechanism. It was calculated as shown below:

$$ b=\left(-3.6167\right)-(0.0160)\left(\mathrm{NISS}\right)-(1.1358)\left(\mathrm{AgeIndex}\right)+(0.2671)\left(\mathrm{RRscore}\right)+(0.8206)\left(\mathrm{SBPscore}\right)+(0.6071)\left(\mathrm{GCSscore}\right) $$

Physiological parameters used in RTS were calculated using ambulance service records, medical records from the transferring hospitals, and triage counter to identify the best physiologic initial values. Best efforts were made in the process of data collection to maintain data accuracy. In-hospital mortality was used as the study’s primary end point. The data collected were analyzed using Version 21.0. Inc. IBM Corp. Chicago: SPSS*.*

### Statistical analysis

Areas under the receiver operating characteristic (AUC) curve were computed to examine the predictive ability of each trauma score for death. All comparisons were analyzed using the MedCalc. A *p* value < 0.05 was considered statistically significant.

Additionally, the Youden index and associated optimal cutoff values for each scoring system were calculated to predict mortality [20]. The corresponding accuracy, positive predictive value, and negative predictive value were obtained. Youden index is regarded as the difference between the true positive rate (sensitivity) and the false positive rate (1-specificity). Maximizing this index allows to find, from the receiver operating characteristics (ROC) curve, an optimal cutoff point. It is defined as the vertical distance between the ROC curve and the first bisector line (or diagonal line) [21, 22].

## Results

A total of 2208 trauma patients with mean age of 36 (SD = 16) years were enrolled. Amongst the trauma patients, 90.5% were blunt trauma with a mortality rate of 10.8%. The characteristic of the study population is as illustrated in (Table 2).

**Table 2:** Characteristics of the study population

	Total study cohort Mean (SD) or *n* (%)
*N*	2208
Age (years)	36 (16)
Sex	
Male	1957 (88.6%)
Female	251 (11.4%)
Mechanism	
Blunt	1999 (90.5%)
Penetrating	204 (9.2%)
Blast	5 (0.2%)
NISS	19.39 (14.01)
RTS	7.384 (0.994)
MTOS-TRISS	0.917 (0.131)
NTrD-TRISS	0.893 (0.115)

*NISS* New Injury Severity Score, *RTS* Revised Trauma Score, *TRISS* Trauma and Injury Severity Score, *MTOS* Major Trauma Outcome Study, *NTrD* National Trauma Database

**Fig. 1:**
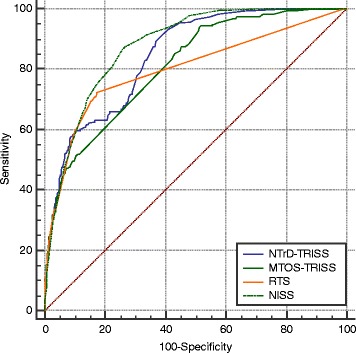
Receiver operating characteristic (ROC) curves of New Injury Severity Score (NISS), Revised Trauma Score (RTS), MTOS-Trauma and Injury Severity Score (TRISS) and NTrD-TRISS in predicting trauma mortality in the study population. *MTOS*, Major Trauma Outcome Study; *NTrD*, National Trauma Database

The AUCs of NISS, RTS, MTOS-TRISS, and NTrD-TRISS were 0.878, 0.802, 0.812, and 0.848, respectively (Fig. 1 and Table 3). The predictive ability of the NISS score were significantly better than RTS, MTOS-TRISS, and NTrD-TRISS (*p* < 0.001) (Table 3).

**Table 3:** Areas under the receiver operating characteristic (ROC) curve of each scoring system in predicting trauma mortality and pairwise comparison between the scores

Scoring system	AUC	95% CI	Pairwise comparison			
			NISS (*P* value)	RTS (*P* value)	MTOS-TRISS (*P* value)	NTrD-TRISS (*P* value)
NISS	0.878	0.864 to 0.892	–	< 0.001	< 0.001	0.0126
RTS	0.802	0.785 to 0.818	< 0.001	–	0.479	0.0014
MTOS-TRISS	0.812	0.795 to 0.828	< 0.001	0.479	–	0.0002
NTrD-TRISS	0.848	0.833 to 0.863	0.0126	0.0014	0.0002	–

*AUC* area under the receiver operating characteristic, *CI* confidence interval, *NISS* New Injury Severity Score, *RTS* Revised Trauma Score, *TRISS* Trauma and Injury Severity Score, *MTOS* Major Trauma Outcome Study, *NTrD* National Trauma Database

The cutoff value of the NISS was 24, with a sensitivity of 86.6% and specificity of 74.3% (Table 4). The accuracy of mortality prediction was 75.6% with a positive predictive value of 29.02 and negative predictive value of 97.86% (Table 4).

**Table 4:** Optimal cutoff value derived for each scoring system and the corresponding sensitivity, specificity, accuracy, positive, and negative predictive values

Scoring system	Cutoff value	Sensitivity (%)	Specificity (%)	Accuracy (%)	PPV (%)	NPV (%)
NISS	> 24	86.6	74.3	75.63	29.02	97.86
RTS	< 7.81	72.4	82.8	81.7	33.76	96.12
MTOS-TRISS	< 0.961	94.14	48.9	53.8	18.26	98.56
NTrD-MTOS	< 0.934	89.5	63.3	66.13	22.83	98.03

*NISS* New Injury Severity Score, *RTS* Revised Trauma Score, *TRISS* Trauma and Injury Severity Score, *MTOS* Major Trauma Outcome Study, *NTrD* National Trauma Database, PPV positive predictive value, NPV negative predictive value

The cutoff value of the RTS was 7.81. This achieved fairly good sensitivity of 72.4% and specificity of 82.8%. The mortality was predicted with a high accuracy of 81.7%. Both the MTOS-TRISS and NTrD-TRISS revealed high sensitivity but poor specificity and accuracy (Table 4).

## Discussion

Original data is an important building block to establish the foundation needed to improve treatment modalities of a young trauma center. By identifying the best tool to predict injury severity and trauma death, it allows the local hospital setup to provide accurate triaging, allocation of resources and overall improvement by service quality comparison.

When an AUC value from ROC analysis is above 0.8, the score is considered to be a good predictive tool [23]. AUC value of NISS was 0.878 which indicates good predictive ability for trauma deaths. Similar findings were seen in other publications that the NISS score was superior in predicting trauma mortality, more so in cases of blunt trauma and firearm injuries [16, 24].

Accurate calculation of a new injury severity score is possible only in the presence of reliable test findings, surgery, or autopsy results. There is a limitation in determining injury severity in an emergency setting [9]. To overcome this limitation, organ injury severity assessment of each patient in our study was performed by a surgeon following full evaluation on the imaging, operative, and autopsy findings. All discrepancies were discussed prior to data entry. This reduces data entry error and improves the accuracy of our trauma surgery database.

NISS score mainly uses anatomical grading to determine injury severity and predict death. Relative difference to NISS, the RTS, and TRISS scores uses physiological parameters to determine the probability of death. RTS and TRISS scores incorporate respiratory rate and systolic blood pressure, which has a vast range of fluctuation depending on the time of first recorded reading [14]. Our results revealed that RTS, MTOS-TRISS, and NTrD-TRISS scores had fairly equal AUC values in comparison to NISS score in predicting trauma death. RTS had better accuracy when compared to NISS despite its lower AUC value. This may be attributed to the maximal effort made to ensure accurate recording of the first physiological parameters before any major medical intervention was performed.

TRISS is a combined anatomic and physiologic scoring system. It has shown to be a good prediction score but with multiple inconsistencies. This may be due to its use of physiologic indices [25] and inappropriate inclusion of coefficients which were originally developed from MTOS [26]. The coefficients from MTOS might not be a true representation of the population in other country where the trauma system is not yet well developed. New coefficients derived from Malaysia National Trauma Database by a team of emergency physicians from a local Malaysian population may increase the predictive ability of the TRISS score [18].

A trauma scoring system is an important step to identify patients at high risk of death. It allows accurate triaging of severely injured patients. To create an ideal scoring system that suits the population of a developed country which can be used universally in all populations is challenging. Differences in body build (BMI, physiology) in a western population where the trauma scores were being devised may differ from an Asian population. Therefore, we compare the existing trauma scoring systems, i.e., the NISS, RTS, and TRISS in Malaysia (an upper middle income Asian country). Promising results show that these scoring systems are sensitive and predictive in assessing trauma death in Malaysia (a country where the trauma system is at the developing stage).

## Conclusion

Amongst the four scores, the NISS score has the highest predictive ability to determine trauma mortality in our regional Malaysian trauma center. However, a joint research with new trauma centers in Malaysia is needed to validate this scoring system.

## Abbreviations

AUC: Area under the receiver operating characteristic
CI: Confidence interval
ICU: Intensive care unit
ISS: Injury Severity Score
MTOS: Major Trauma Outcome Study
NISS: New Injury Severity Score
NTrD: National Trauma Database
ROC: Receiver operating characteristic
RTS: Revised Trauma Score
TRISS: Trauma and Injury Severity Score
